# Effects of Cilostazol and Isosorbide Mononitrate on Cerebral Hemodynamics in the LACI-1 Randomized Controlled Trial

**DOI:** 10.1161/STROKEAHA.121.034866

**Published:** 2021-12-01

**Authors:** Gordon W. Blair, Esther Janssen, Michael S. Stringer, Michael J. Thrippleton, Francesca Chappell, Yulu Shi, Iona Hamilton, Katie Flaherty, Jason P. Appleton, Fergus N. Doubal, Philip M. Bath, Joanna M. Wardlaw

**Affiliations:** Brain Research Imaging Centre, Centre for Clinical Brain Sciences, UK Dementia Institute Centre at the University of Edinburgh, United Kingdom (G.W.B., M.S.S., M.J.T., F.C., Y.S., I.H., F.N.D., J.M.W.).; Department of Neurology, Donders Institute for Brain, Cognition and Behaviour, Donders Centre for Medical Neuroscience, Radboud University Medical Center, Nijmegen, the Netherlands (E.J.).; Stroke Trials Unit, Division of Clinical Neuroscience, University of Nottingham, United Kingdom (K.F., J.P.A., P.M.B.).; Stroke, University Hospitals Birmingham NHS Foundation Trust, Queen Elizabeth Hospital, Mindelsohn Way, United Kingdom (J.P.A.).; Stroke, Queen’s Medical Centre Campus, Nottingham University Hospitals NHS Trust, United Kingdom (P.M.B.).

**Keywords:** cerebral small vessel diseases, cilostazol, cognitive dysfunction, ischemic stroke, magnetic resonance imaging

## Abstract

Cerebral small vessel disease—a major cause of stroke and dementia—is associated with cerebrovascular dysfunction. We investigated whether short-term isosorbide mononitrate (ISMN) and cilostazol, alone or in combination, improved magnetic resonance imaging–measured cerebrovascular function in patients with lacunar ischemic stroke.

**Methods:**

Participants were randomized to ISMN alone, cilostazol alone, both ISMN and cilostazol, or no medication. Participants underwent structural, cerebrovascular reactivity (to 6% carbon dioxide) and phase-contrast pulsatility magnetic resonance imaging at baseline and after 8 weeks of medication.

**Results:**

Of 27 participants (mean age, 68±7.7; 44% female), 22 completed cerebrovascular reactivity and pulsatility imaging with complete datasets. White matter cerebrovascular reactivity increased in the ISMN (β=0.021%/mm Hg [95% CI, 0.003–0.040]) and cilostazol (β=0.035%/mm Hg [95% CI, 0.014–0.056]) monotherapy groups and in those taking any versus no medication (β=0.021%/mm Hg [95% CI, 0.005–0.037]).

**Conclusions:**

While limited by small sample size, we demonstrate that measuring cerebrovascular function with magnetic resonance imaging is feasible in clinical trials and that ISMN and cilostazol may improve cerebrovascular function.

**Registration:**

URL: https://www.clinicaltrials.gov; Unique identifier: NCT02481323. URL: www.isrctn.com; Unique identifier: ISRCTN12580546. URL: www.clinicaltrialsregister.eu; Unique identifier: EudraCT 2015-001953-33.

## Abstract 2

Supplemental Digital Content is available in the text.

Cerebral small vessel disease (SVD) is a major cause of stroke and dementia. No specific treatment exists to stop disease progression.^1^

SVD is associated with reduced cerebrovascular reactivity (CVR^2^; the ability of blood vessels to increase blood flow), more pulsatile blood flow, and impaired cerebrospinal fluid (CSF) dynamics.^2,3^

Isosorbide mononitrate (ISMN) and cilostazol have pharmacological effects that could improve these cerebrovascular dysfunctions.^1^ Cilostazol can reduce stroke recurrence^4^ and may reduce cognitive impairment.^5^ Glyceryl trinitrate—a shorter-acting nitrate than ISMN—improved cognitive scores in some patients treated early after ischemic stroke.^6^

We measured CVR, cerebral blood flow, and CSF dynamics in patients with lacunar stroke randomly assigned to receive ISMN or cilostazol monotherapy, combination therapy, or avoid these medications in the LACI-1 trial (LACunar Intervention-1).^7^

## Methods

### Data Availability Statement

Access requests can be submitted to the corresponding author.

### Participants and Assessments

This study was a substudy of the main LACI-1 trial. LACI- 1 was a phase IIa, partial factorial, prospective randomized open-label blinded end point trial. Our methods are published.^7^ We recruited patients with nondisabling lacunar ischemic stroke.

All participants were scanned at randomization and at week 8 in the cilostazol monotherapy, ISMN monotherapy, and ISMN and cilostazol groups and week 3 in the no-medication group (after which this group commenced cilostazol and ISMN to test the alternative order of starting dual therapy). Medication compliance, side effects, and blood pressure (BP) were also assessed at each visit.

All participants provided written informed consent. Ethical approval was obtained from the Scotland-A Research Ethics Committee (Ref: 15/SS/0154).

### Intervention

Participants were randomized to ISMN monotherapy, cilostazol monotherapy, combination ISMN, and cilostazol or no medication.^7^ Dose was titrated to ISMN 25 mg BID and cilostazol 100 mg BID.^7^ Medication was taken for 8 weeks. Participants were masked to treatment allocation, and investigators assessing outcomes including all image analysis were blinded to treatment allocation.^7^

### Imaging

We performed brain scanning using a 1.5T GE MRI scanner (SignaHDxt; General Electric, Milwaukee, WI). Structural sequences included T1-weighted, T2-weighted, fluid attenuated inversion recovery, and gradient recalled echo.

### CVR Acquisition

During a 12-minute blood oxygen level–dependent magnetic resonance imaging (MRI) scan, participants alternated between breathing medical air and 6% carbon dioxide (CO_2_) in air, as is published previously.^8^

### Pulsatility Acquisition

Our pulsatility method is published previously.^2,3^ We used a 2-dimensional cine phase-contrast sequence to measure flow in the internal carotid and vertebral arteries, superior sagittal, straight, and transverse venous sinuses, and foramen magnum and aqueduct CSF flow.

### Image Analysis

We performed CVR analysis by regressing blood oxygen level–dependent signal against end-tidal CO_2_, with CVR expressed as %blood oxygen level–dependent signal change/mm Hg change in end-tidal CO_2_ as published previously.^7,8^ We extracted regional measurements of CVR in identical regions of the same anatomic structures at the two time points. Image analysis was performed by investigators blinded to treatment allocation.

Structural and pulsatility analysis methods are pulished.^2,3^ Pulsatility index (PI) was calculated as (Flow_maximum_−Flow_minimum_)/Flow_mean_.

### Statistical Analysis

We used R, version 1.0.143, for statistical analyses. To examine the effect of treatment, 2 separate analyses were done. First, an intention-to-treat analysis (main article) and second, a per-protocol analysis (Supplemental Material) where participants who had stopped taking medication at the time of the follow-up scan were excluded from the treatment groups and analyzed as being in the no-medication group. We used multiple linear regression to assess the effect of treatment groups and being on any versus no medication on changes in white matter (WM) and gray matter CVR, PI, and CSF flow adjusting for age because this is suggested to affect CVR. This analysis was repeated and adjusted for systolic BP in place of age to examine the confounding effect of BP. To assure normality of the residuals and assess heteroscedasticity, we examined QQ plots, histograms of residuals, and plots of residual versus fitted values to examine.

This analysis was repeated and adjusted for systolic BP in place of age to examine the confounding effect of BP. Additional analyses were also performed controlling for systolic BP change in place of age between baseline and follow-up.

## Results

Twenty-seven of the 28 participants recruited to LACI-1 consented to the MRI substudy. Full CVR data sets were obtained in 23 participants and full pulsatility data sets in 24 (Supplemental Material). The Figure shows the CONSORT flowchart.

**Figure.**
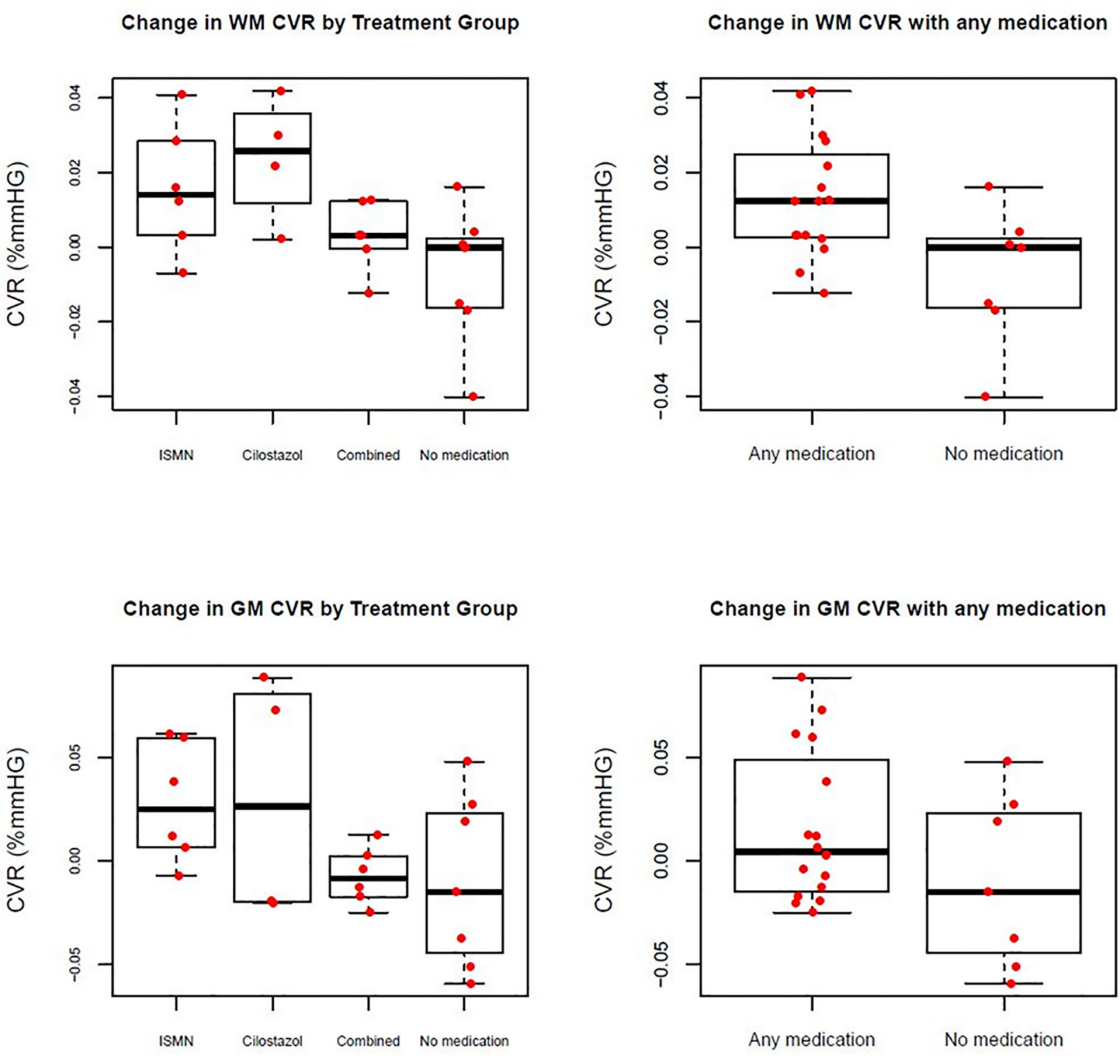
**Change in cerebrovascular reactivity (CVR) by treatment group and any vs no medication.** GM indicates gray matter; ISMN, isosorbide mononitrate; and WM, white matter.

The mean age of included participants was 68±7.7 years (range, 53–83 years), Table 1.

**Table 1.**
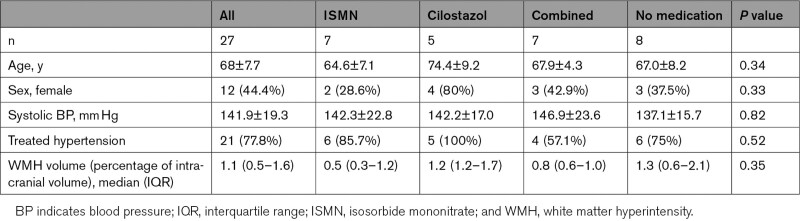
Patient Characteristics

### CVR Change After Treatment

WM CVR increased with ISMN and cilostazol monotherapy (both *P*<0.05) and in participants taking any versus no trial drug (*P*<0.05) but not combination therapy (Table 2).

**Table 2.**
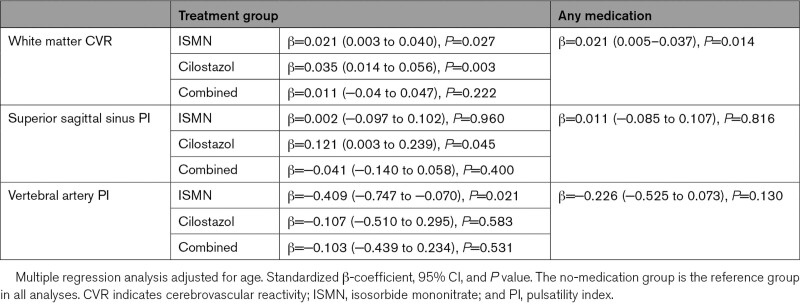
Associations Between Change in White Matter CVR, Intracranial Pulsatility, and Treatment

There was no increase in gray matter CVR.

### Pulsatility Change After Treatment

Superior sagittal sinus PI increased with cilostazol alone. Vertebral artery PI decreased with ISMN alone (Table 2). CSF pulsatility did not change.

## Discussion

Over an 8-week period, treatment with ISMN or cilostazol alone, or any drug versus no drug, increased WM but not gray matter CVR. Effects on pulsatility varied: ISMN decreased PI in the vertebral arteries; however, cilostazol increased superior sagittal sinus PI, which could be secondary to cilostazol increasing heart rate.^7^ No changes in CSF flow dynamics were detected. Combination therapy did not have the effect of monotherapy although CVR in WM increased in participants taking any versus no medication, which includes those allocated dual therapy. Small numbers in each treatment group and participants finding it difficult to reach maximum dose of both drugs in combination means this is more likely sample size related and the true difference between effects of monotherapy versus combination remains undetermined.

Prior studies have measured medication effects on resting cerebral blood flow in similar patient populations. The DANTE study (Discontinuation of Antihypertensives in the Elderly) showed no change in MRI-measured cerebral blood flow after antihypertensive medication withdrawal, compared with continuing antihypertensives.^9^ The PRESERVE trial (Prevention of Serious Adverse Events Following Angiography) showed no change in MRI-measured cerebral blood flow with more versus less intensive BP lowering in patients with stroke-related moderate-to-severe SVD.^10^ Others have demonstrated pravastatin, atorvastatin, perindopril, and vinpocetine all increase transcranial Doppler-measured vasoreactivity.^11–15^ The changes in CVR we demonstrated were independent of any change in systolic BP induced by medication, adding further evidence that changes in blood flow and vascular function in older individuals with SVD have a complex relationship with BP. We have previously demonstrated stronger associations of WM than gray matter CVR with SVD.^2^ WM may be differentially more impaired in SVD and thus more amenable to a detectable pharmacological improvement.

Lack of improvement in PI could reflect that modification of vascular stiffening requires longer treatment. Cilostazol has previously been shown to decrease PI after 90 days in patients with lacunar infarction.^16^

### Limitations

The sample is small. However, a primary aim of LACI-1 was to establish feasibility of the drug regimen to inform a larger trial (LACI-2; ISRCTN14911850) and secondarily to gather efficacy and safety data.^7,17^

The randomization was imperfect, as in the overall main trial,^7^ with the cilostazol-only group being older.

### Conclusions

We demonstrated feasibility of cerebrovascular function MRI in a clinical trial of lacunar ischemic stroke and SVD and detected changes in CVR and pulsatility that support the positive modification of cerebrovascular function by existing medications. Larger studies over longer time periods will assess whether these improvements translate into clinical benefits.

## Article Information

### Acknowledgments

We thank the participants and members of the trial committees.

### Sources of Funding

Alzheimer’s Society Ref: 252 (AS-PG-14-033). European Union Horizon 2020 project No. 666881, SVDs@Target (G.W. Blair Dr Stringer); Stroke Association Princess Margaret Research Development Fellowship (G.W. Blair); Stroke Association Garfield Weston Foundation Senior Clinical Lectureship (F.N. Doubal); National Health Service Research Scotland (F.N. Doubal); China Scholarship Council/University of Edinburgh (Dr Shi); National Health Service Lothian Research & Development Office (Dr Thrippleton); Row Fogo Charitable Trust; Scottish Funding Council via Scottish Imaging Network, A Platform for Scientific Excellence Collaboration; Fondation Leducq (ref. 16CVD05); Edinburgh and Lothians Health Foundation; National Institute for Health Research (NIHR) Health Technology Assessment (HTA) TARDIS Trial (Dr Appleton and P.M. Bath); British Heart Foundation RIGHT-2 Trial (Dr Appleton and P.M. Bath); NIHR HTA TICH-2 trial (Tranexamic Acid for Hyperacute Primary Intracerebral Haemorrhage; K. Flaherty). P.M. Bath is Stroke Association Professor of Stroke Medicine and Emeritus NIHR Senior Investigator. UK Dementia Research Institute Centre at the University of Edinburgh (funded by the Medical Research Council, Alzheimer’s Society and Alzheimer’s Research UK; Dr Wardlaw).

### Disclosures

P.M. Bath is on the advisory boards of Sanofi, Nestle, DiaMedica, Moleac, Phagenesis, and ReNeuron. The other authors report no conflicts.

### Supplemental Material

LACI-1 Trial Inclusion and Exclusion Criteria

Supplemental Methods

CONSORT Flowcharts

Expanded Participant Characteristics Table

Expanded Intention-to-Treat Results Table

Per-Protocol Analyses

Summary Table

Expanded Acknowledgments

CONSORT Checklist

## Supplementary Material


