# Supplementary material for: Effects of Cilostazol and Isosorbide Mononitrate on Cerebral Hemodynamics in the LACI-1 Randomized Controlled Trial
Source: Stroke. 2021 Dec 1;53(1):29–33. doi: 10.1161/STROKEAHA.121.034866 (PMC8700302; doi:10.1161/STROKEAHA.121.034866)
Supplement: Supplementary file 1 [file str-53-029-s001.pdf]

Supplementary methods

S1) LACI-1 Trial Inclusion and Exclusion Criteria

S2) Supplementary methods

S3) CONSORT flow charts including reasons for loss of data and medication compliance

S4) Expanded Participants Characteristics Table

S5) Expanded Intention to Treat Results Table

S6) Per protocol analyses

S7) Summary table of review of literature on effects of drugs on transcranial Doppler assessed CVR

S8) Expanded Acknowledgements

S9) CONSORT Checklist

S1: LACI-1 Trial Inclusion and Exclusion Criteria

Inclusion Criteria	Exclusion Criteria
1. Mild symptomatic ischemic lacunar stroke in the past four years, compatible with a clinical lacunar stroke syndrome, with brain MRI or CT scanning showing a symptomatic small subcortical (lacunar) infarct (<20 mm), or if no recent relevant infarct is visible, that excluded other cause for symptoms. Clinical or imaging evidence of a prior non-lacunar stroke is not an exclusion as long as the randomizing clinician is confident that the non-lacunar stroke is not responsible for the index lacunar stroke symptoms.<br>2. Age 35 years.<br>3. Independent in activities of daily living (modified Rankin Scale of ≤2) and able to give informed consent.	1. Other significant neurological illness since the incident stroke.<br>2. Age<35 years.<br>3. Montreal Cognitive Assessment (MoCA)<20.<br>4. Requiring assistance with activities of daily living (modified Rankin Scale≥3).<br>5. Active cardiac disease.<br>6. Carotid stenosis>50% (NASCET criteria) on the side of the symptomatic stroke lesion requiring urgent intervention. Note: successfully treated carotid artery stenosis may be included.<br>7. Definite indication for, or contraindication to, cilostazol or ISMN.<br>8. Unable to swallow.<br>9. Bleeding tendency.<br>10. Unlikely to comply with trial medication based on past history or lifestyle.<br>11. Planned surgery during the trial period.<br>12. History of intracranial haemorrhage (but not<br>asymptomatic haemorrhagic transformation of an infarct).<br>13. Other life-threatening illness.<br>14. History of drug overdose, attempted suicide or significant active mental illness.<br>15. Pregnant or breastfeeding women.<br>16. Women of childbearing age not taking contraception.<br>17. Use of prohibited medications (anticoagulants, phosphodiesterase 5' inhibitors, macrolides, ketoconazole, itraconazole, omeprazole).<br>18. Creatinine clearance<25 ml/min.<br>19. Hepatic impairment.<br>20. Current enrolment in another Clinical Trial of Investigational Medicinal Product (CTIMP).

S2 Supplementary methods

Trial Design

LACI-1 was a Phase IIa, partial factorial, prospective, randomised, open-label, blinded endpoint (PROBE) trial. The main trial was conducted in Edinburgh and Nottingham. The imaging substudy was conducted in Edinburgh only. The first participant was randomised on 16th March 2016, the final participant on 29th August 2017 and final follow-up was completed on 31 October 2017.

The primary outcome of the main trial was the proportion of participants achieving target dose by the end of the eight-week trial period[7]. The results reported here for effects of cilostazol and ISMN on CVR, cerebral arterial and venous pulsatility and CSF flow dynamics were pre-specified secondary outcomes. Additional secondary outcomes, including adverse events, have been reported previously[7,17].

Participants and assessments

Our methods are published[7]. We recruited patients with symptomatic lacunar ischaemic stroke in the past four years. Participants were over 35 years of age and functionally independent.

All participants were scanned at randomisation. Participants assigned to a medication group were then scanned at week 8, participants assigned to the no medication group were scanned at week 3 (participants in this group then commenced medication for the remainder of the main trial).

All participants provided written informed consent. Ethical approval was obtained from the Scotland A Research Ethics Committee (Ref 15/SS/0154).

Intervention

Participants were randomised, using a web-based system utilising minimisation on age, BP, time since stroke and SVD severity, at a 1:1:1:1 ratio to ISMN monotherapy, cilostazol monotherapy, combination ISMN and cilostazol or no medication[7]. Dose was titrated, as tolerated, over three weeks to ISMN 25mg twice daily and cilostazol 100mg twice daily[7]. Medication was taken for eight weeks. Participants were masked to treatment allocation and study staff assessing outcomes were blinded to treatment allocation[7].

Imaging

We performed brain scanning using a 1.5 Tesla GE MRI scanner (SignaHDxt, General Electric, Milwaukee, WI) at the University of Edinburgh's Brain Research Imaging Centre, Western General Hospital, Edinburgh.

3D T1-weighted, axial T2-weighted, axial fluid attenuated inversion recovery (FLAIR), and gradient echo sequences were acquired using an 8-channel phased array head coil. BOLD scanning was performed to assess CVR at 4mm isotropic resolution as described previously[8]. Phase contrast MRI assessed flow and pulstility in the cervical arteries, intracranial venous sinuses and subarachnoid space as described previously[2,3].

CVR acquisition

Our CVR procedure and its development are published in detail[8]. Briefly patients wore an anaesthetic face mask attached to a bespoke unidirectional breathing circuit (Intersurgical, Wokingham, UK). Monitoring equipment recorded pulse rate, oxygen saturation, blood pressure (Millennia 3155A and Magnitude 3150 MRI; Invivo, Best, The Netherlands) and end-tidal carbon dioxide (EtCO2; AEI Technologies, Pittsburgh, USA) throughout the examination. During a 12-minute BOLD MRI scan patients alternated between breathing medical air and 6% carbon dioxide (CO2) in air (BOC Special Products, UK). The full paradigm comprised two minutes air, three minutes CO2, two minutes air, three minutes CO2, two minutes air, end of scan.

Pulsatility acquisition

Details of our pulsatility imaging are described previously[2,3]. Briefly, we used a 2D cine phase-contrast sequence with retrospective peripheral pulse gating to acquire 32 velocity images per cardiac cycle in slices located to measure flow in the internal carotid and vertebral arteries, superior sagittal, straight and transverse venous sinuses and CSF flow across the cerebral aqueduct and foramen magnum.

Image Processing and Analysis

Structural image analysis:

An expert neuroradiologist rated the structural imaging obtained at randomisation and at follow-up for WMH, enlarged perivascular spaces, lacunes and microbleeds using validated visual ratings according to the STRIVE criteria. All analyses were blinded to the patient's clinical data, treatment allocation and to the CVR results.

For quantitative analysis each subject's structural images were co-registered to the T2 space. We calculated WMH volumes using a validated semi-quantitative technique described previously[2,3].

CVR Image Analysis:

We performed CVR image processing and analysis as described previously[8] with additional steps to ensure measurement of CVR in the identical regions of the same anatomical structures at the two timepoints.

Briefly, to generate voxel-wise CVR maps, we regressed BOLD signal against EtCO2 with CVR expressed as %BOLD signal change/mmHg change in EtCO2. We realigned BOLD images (using SPM 8) to the mean BOLD image prior to determining the transformation between mean BOLD and visit T2W image spaces (using FSL FLIRT). Subject-specific templates based on the registered T1W image were created (using ANTs) to generate an unbiased space for delineating regions of interest. We then manually drew three subcortical grey matter (thalamus, putamen, caudate head) and four subcortical white matter (frontal, posterior, periventricular and centrum semiovale) regions of interest on the template before registering back to the visit specific T2 space. The regions of interest were thresholded at 0.5 and dichotomised before inspection to ensure they were within the

expected region. Voxels that were part of large blood vessels or the patient's stroke lesion were manually excluded. The regions of interest were then registered to the BOLD images (using FSL FLIRT). The resultant CVR values for all grey and white matter regions were also averaged for the participant to give a grey matter and white matter CVR value per participant.

Pulsatility Image Analysis:

Analysis of pulsatility imaging has been described previously[2,3]. Regions of interest were drawn around the relevant vessels. For bilateral structures we calculated sum flow and mean velocities. Total cerebral blood flow was calculated as the sum of ICA and VA flow and normalized to brain volume expressed as mL/min/100mL brain tissue. Pulsatility index (PI) in each structure was calculated as (Flowmaximum − Flowminimum)/Flowmean; resistivity index (RI) was calculated as (Flowmaximum − Flowminimum)/Flowmaximum, with higher values indicating more pulsatile or more resistive blood flow. CSF flow defined the net flow in the aqueduct and foramen magnum, respectively. CSF stroke volume reflects the total volume of CSF flow per cardiac cycle and is calculated by averaging the absolute cranial and caudal flow across the cardiac cycles.

S4a: CONSORT Diagram

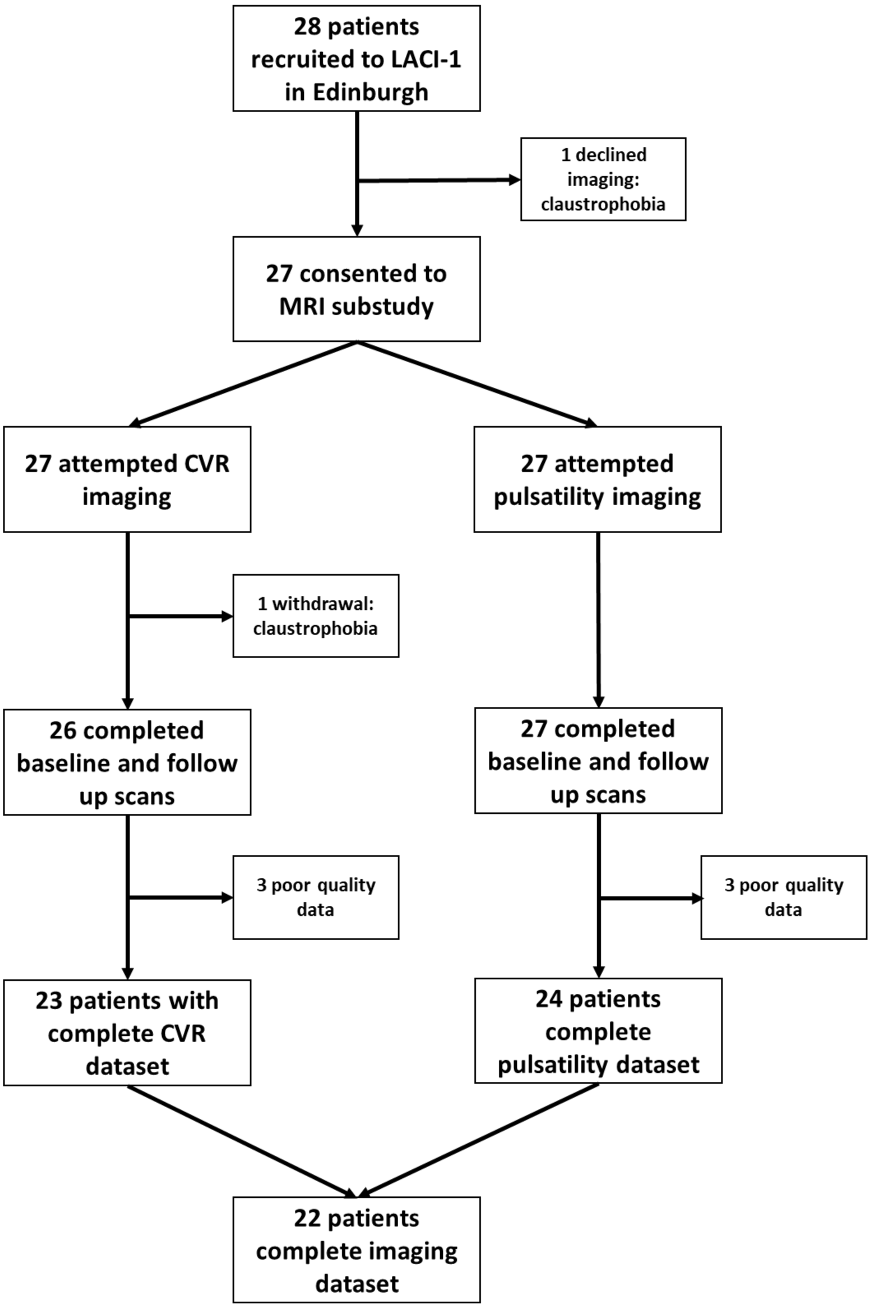

S4b: CVR and Pulsatility Imaging CONSORT diagrams with reasons for exclusion and compliance with medication.

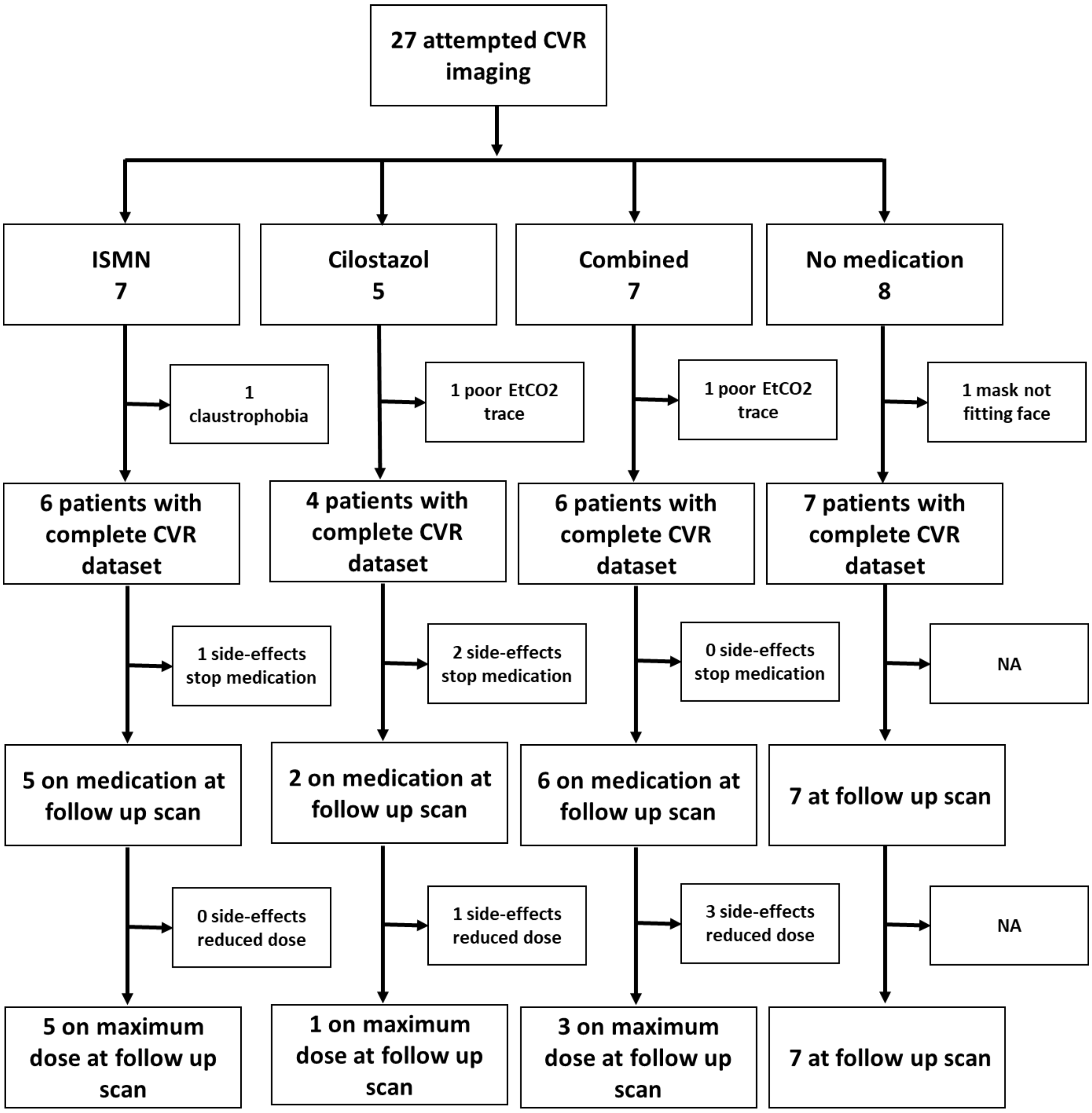

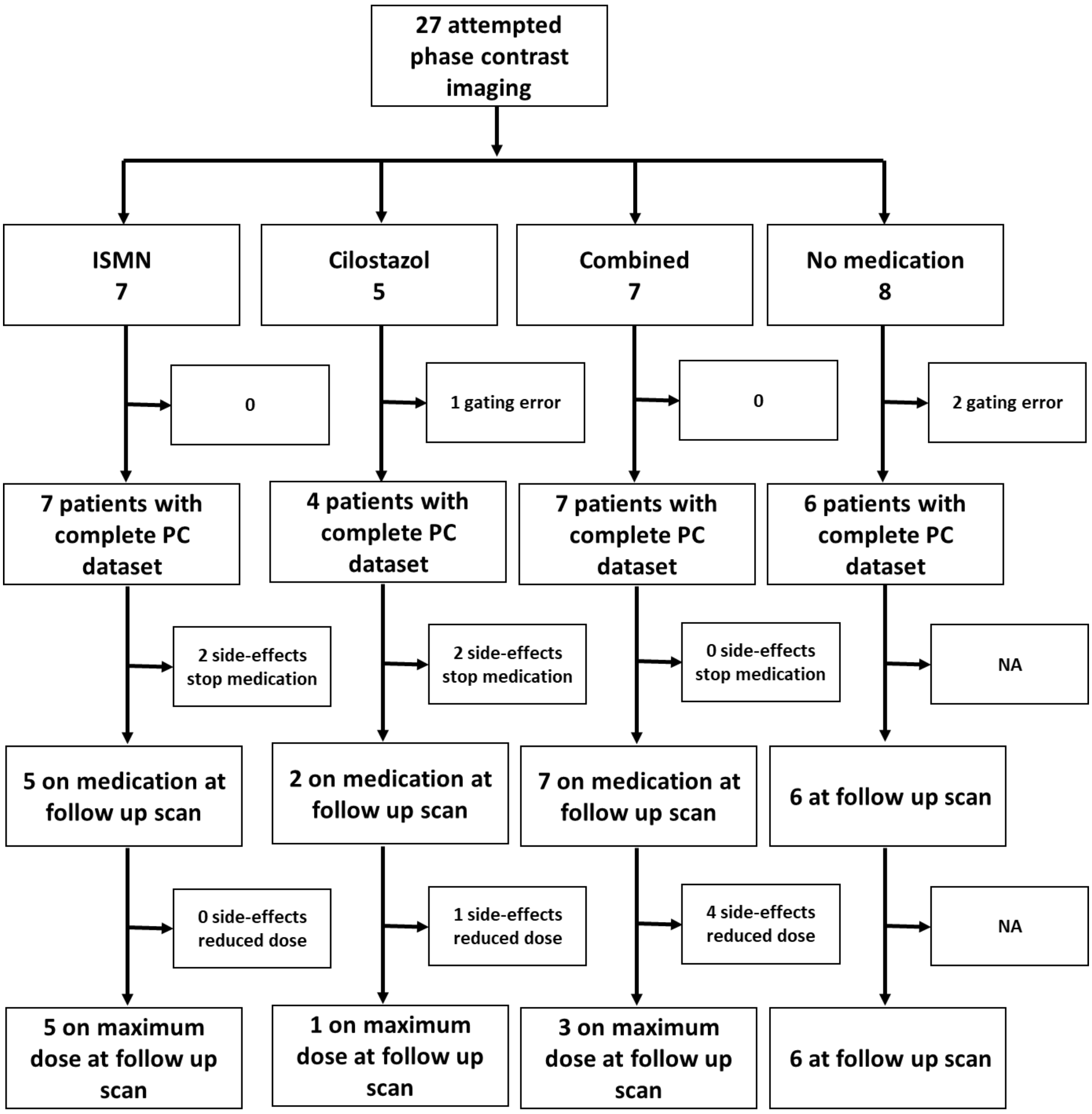
27 attempted phase contrast imaging
ISMN 7
Cilostazol 5
Combined 7
No medication 8
0
1 gating error
0
2 gating error
7 patients with complete PC dataset
4 patients with complete PC dataset
7 patients with complete PC dataset
6 patients with complete PC dataset
2 side-effects stop medication
2 side-effects stop medication
0 side-effects stop medication
NA
5 on medication at follow up scan
2 on medication at follow up scan
7 on medication at follow up scan
6 at follow up scan
0 side-effects reduced dose
1 side-effects reduced dose
4 side-effects reduced dose
NA
5 on maximum dose at follow up scan
1 on maximum dose at follow up scan
3 on maximum dose at follow up scan
6 at follow up scan

S5: Expanded Participants Characteristics Table

	All	ISMN	Cilostazol	Combined	No Medication
Patients (number)	27	7	5	7	8
Age (years)	68 ± 7.7	64.6 ± 7.1	74.4 ± 9.2	67.9 ± 4.3	67.0 ± 8.2
Sex, female	12 (44.4%)	2 (28.6%)	4 (80%)	3 (42.9%)	3 (37.5%)
Onset to randomisation (days)	247.8±289.9	214.8±295.7	465.2 ±353.2	197.6±283.6	184.6±234.7
Systolic BP (mmHg)	141.9 ± 19.3	142.3 ± 22.8	142.2 ± 17.0	146.9 ± 23.6	137.1 ± 15.7
Diastolic BP (mmHg)	79.7 ± 9.6	80.1 ± 10.2	80.6 ± 13.9	79.6 ± 6.2	79.0 ± 10.5
Treated hypertension	21 (77.8%)	6 (85.7%)	5 (100%)	4 (57.1%)	6 (75%)
Treated hyperlipidaemia	23 (85.2%)	6 (85.7%)	5 (100%)	5 (71.4%)	7 (87.5%)
Diabetes	2 (7.4%)	0	0	1 (14.3%)	1 (12.5%)
WMH volume (% of intracranial volume) median (IQR)	1.1 (0.5 – 1.6)	0.5 (0.3 – 1.2)	1.2 (1.2 – 1.7)	0.8 (0.6 – 1.0)	1.3 (0.6 – 2.1)
Periventricular Fazekas score (median)	2	1	2	2	2
Deep white matter Fazekas score (median)	1	1	2	2	1.5
Basal ganglia PVS score (median)	2	2	2	3	1
Centrum Semiovale PVS score (median)	3	3	3	3	2
Presence of lacunes	19 (70.4%)	3 (42.9%)	4 (80%)	5 (71.4%)	7 (87.5%)
Presence of microbleeds	5 (20.8%)	1 (14.3%)	1 (25%)	2 (33.3%)	1 (14.3%)
White Matter CVR at Baseline (%/mmHg)	0.05 ± 0.02	0.04 ± 0.02	0.05 ± 0.01	0.04 ± 0.04	0.05 ± 0.02
White Matter CVR at Follow Up (%/mmHg)	0.05 ± 0.02	0.06 ± 0.01	0.06 ± 0.02	0.05 ± 0.01	0.04 ± 0.02
Grey Matter CVR at Baseline (%/mmHg)	0.15 ± 0.05	0.14 ± 0.04	0.16 ± 0.03	0.15 ± 0.08	0.15 ± 0.05
Grey Matter CVR at Follow Up (%/mmHg)	0.16 ± 0.06	0.17 ± 0.04	0.17 ± 0.06	0.17 ± 0.07	0.13 ± 0.07

Mean ± standard deviation is used for except where stated

S6: Expanded Intention to Treat Results Table

	Treatment group		Any medication
White Matter CVR	ISMN	β=0.021 (0.003–0.040) **p=0.027**	β=0.021 (0.005–0.037) **p=0.014**
	Cilostazol	β=0.035 (0.014-0.056) **p=0.003**	
	Combined	β =0.011 (-0.04 –0.047) p=0.222	
Deep Grey Matter CVR	ISMN	β=0.036 (-0.008–0.081) p=0.103	β=0.026 (-0.01 –0.063) p=0.162
	Cilostazol	β=0.044 (-0.007–0.095) p=0.089	
	Combined	β=0.003 (-0.041–0.047) p=0.886	
Superior sagittal sinus	ISMN	β=0.002 (-0.097-0.102) p=0.960	β=0.011 (-0.085-0.107) p=0.816
	Cilostazol	β=0.121 (0.003-0.239) **p=0.045**	
	Combined	β=-0.041 (-0.140-0.058) p=0.400	
Straight sinus	ISMN	β=-0.058 (-0.204-0.088) p=0.420	β=-0.058 (-0.184-0.068) p=0.349
	Cilostazol	β=0.035 (-0.139-0.208) p=0.679	
	Combined	β=-0.110 (-0.254-0.036) p=0.133	
Transverse sinus	ISMN	β=0.042 (-0.109-0.193) p=0.569	β=0.030 (-0.106-0.166) p=0.652
	Cilostazol	β=0.145 (-0.034-0.324) p=0.107	
	Combined	β=-0.045 (-0.195-0.105) p=0.538	
Internal Carotid Artery	ISMN	β=-0.037 (-0.341–0.268) p=0.803	β=-0.075 (-0.330-0.181) p=0.550
	Cilostazol	β=-0.252 (-0.613-0.110) p=0.161	
	Combined	β=-0.171 (-0.320-0.286) p=0.907	
Vertebral Artery	ISMN	β=-0.409 (-0.747--0.070)	β=-0.226 (-0.525-0.073)

		**p=0.021**	p=0.130
	Cilostazol	β=-0.107<br>(-0.510-0.295)<br>p=0.583	
	Combined	β=-0.103<br>(-0.439-0.234)<br>p=0.531	
Internal Jugular Vein	ISMN	β=0.133<br>(-0.516-0.249)<br>p=0.474	β=-0.155<br>(-0.464–0.155)<br>p=0.311
	Cilostazol	β=-0.065<br>(-0.519-0.389)<br>p=0.767	
	Combined	β=-0.225<br>(-0.605-0.155)<br>p=0.231	
Aqueduct CSF	ISMN	β=0.011<br>(-0.001-0.022)<br>p=0.076	β=0.005<br>(-0.006-0.016)<br>p=0.371
	Cilostazol	β=0.011<br>(-0.003-0.025)<br>p=0.129	
	Combined	β=-0.004<br>(-0.016-0.008)<br>p=0.500	
Subarachnoid space CSF	ISMN	β=0.034<br>(-0.104-0.173)<br>p=0.607	β=-0.017<br>(-0.133-0.099)<br>p=0.761
	Cilostazol	β=-0.043<br>(-0.207-0.121)<br>p=0.592	
	Combined	β=-0.057<br>(-0.194-0.081)<br>p=0.398	

Intention-to-treat analysis of association between change in white matter and grey matter CVR and treatment. Multivariate Regression Analysis is adjusted for age. Standardised β co-efficient, 95% confidence interval and p-value are shown. No medication group is used as reference group in all analyses.

S7: Per protocol analyses

		Treatment group	Any medication
Whole WM	ISMN	β = 0.022<br>(0.002-0.042)<br>**p = 0.031**	β = 0.016<br>(-0.0002-0.033)<br>p = 0.0522
	CI	β = 0.040<br>(0.013-0.066)<br>**p = 0.006**	
	Combined	β = 0.006<br>(-0.011-0.024)<br>p = 0.472	
Whole GM	ISMN	β = 0.033<br>(-0.017-0.083)<br>p = 0.182	β = 0.011<br>(-0.027-0.048)<br>p = 0.560
	CI	β = 0.027<br>(-0.040-0.093)<br>p = 0.403	
	Combined	β = -0.008<br>(-0.051-0.036)<br>p = 0.716	

Per protocol analysis of association between change in white matter and grey matter CVR and treatment. Multivariate Regression Analysis is adjusted for age. Standardised β co-efficient, 95% confidence interval and p-value are shown. No medication group is used as reference group in all analyses.

	Treatment group		Any medication
Superior sagittal sinus PI	ISMN	β = -0.085 (-0.195-0.027) p = 0.128	β = -0.071 (-0.154-0.013) p = 0.092
	CI	β = 0.041 (-0.104-0.187) p = 0.560	
	Combined	β = -0.090 (-0.181-0.001) p = 0.052	
Straight sinus PI	ISMN	β = -0.150 (-0.295- -0.006) **p = 0.042**	β = -0.148 (-0.246- -0.049) **p = 0.005**
	CI	β = -0.129 (-0.319-0.061) p = 0.170	
	Combined	β = -0.151 (-0.270- -0.033) **p = 0.015**	
Transverse sinus	ISMN	β = -0.046 (-0.218 – 0.125) p = 0.579	β = -0.066 (-0.190-0.058) p = 0.279
	CI	β = 0.053 (-0.172-0.278) p = 0.626	
	Combined	β = -0.104 (-0.245-0.037) p = 0.139	
Internal Carotid Artery	ISMN	β = 0.037 (-0.309-0.384) p = 0.824	β = 0.040 (-0.200-0.280) p = 0.735
	CI	β = -0.058 (-0.513-0.398) p = 0.793	
	Combined	β = 0.064 (-0.221-0.349) p = 0.647	
Vertebral Artery	ISMN	β = -0.383 (-0.759- -0.008) **p = 0.046**	β = -0.109 (-0.400-0.181) p = 0.443
	CI	β = 0.054 (-0.439-0.547) p = 0.820	
	Combined	β = -0.013 (-0.322-0.296) p = 0.930	
Internal Jugular Vein	ISMN	β = -0.200 (-0.608-0.207) p = 0.316	β = -0.181 (-0.466-0.103) p = 0.199
	CI	β = -0.009 (-0.543-0.526) p = 0.973	
	Combined	β = -0.213 (-0.548-0.123) p = 0.200	

Per protocol analysis of association between change in pulsatility and treatment. Multivariate Regression Analysis is adjusted for age. Standardised β co-efficient, 95% confidence interval and p-value are shown. No medication group is used as reference group in all analyses.

	Treatment group		Any medication
Aqueduct CSF	ISMN	β = 0.011 (-0.002-0.024) p = 0.105	β = -0.001 (-0.011-0.010) p = 0.929
	CI	β = 0.005 (-0.013-0.022) p = 0.568	
	Combined	β = -0.007 (-0.018-0.004) p = 0.181	
Subarachnoid space CSF	ISMN	β = 0.011 (-0.142-0.164) p = 0.883	β = -0.033 (-0.140-0.075) p = 0.536
	CI	β = -0.012 (-0.213-0.188) p = 0.899	
	Combined	β = -0.059 (-0.184-0.067) p = 0.343	

Per protocol analysis of association between change in CSF stroke volume in aqueduct and subarachnoid space and treatment. Multivariate Regression Analysis is adjusted for age. Standardised β co-efficient, 95% confidence interval and p-value are shown. No medication group is used as reference group in all analyses.

S8: Summary table of effects of drugs on transcranial Doppler assessed CVR

Paper	Patients	Test Drug	Stimulus	Effect
Walters 2004[11]	Lacunar stroke (12 patients)	Perindopril 4mg for 2 weeks	Acetazolamide	18.8±10% increase in cerebral vasomotor reactivity
Sterzer 2001[12]	SVD (16 patients, 68±10yrs)	Pravastatin 20mg 2 months	Acetazolamide	13.8% increase in cerebral vasomotor reactivity
Jovanovic 2013[13]	SVD (30 patients, 61.4±11.5yrs)	Vinpocetine 15mg daily for 3 months	Breath holding index	Trend towards improved breath holding index suggesting better reactivity. Significant improvement in MMSE and modified Rankin (note very low numbers)
Pretnar-Oblak 2006[15]	Lacunar infarct (18 patients 61.1±7.6yrs)	Atorvastatin for 3 months	L-arginine	~4% increase in L-arginine reactivity
Sander 2005[14]	Healthy adults (25 participants, age 28.8yrs)	Pravastatin 40mg 14 days	CO2	Vasomotor reserve capacity increased with treatment

S9: Expanded Acknowledgements

Members of Trial Steering Committee in addition to the authors:

Dr John Bamford, Independent Chair; Euan Haig, Sandra Duggan, Kieran Hanna, Mair Graham, participant representatives; and the Trial Sponsor

The LACI-1 Data Monitoring Committee:

Professor Colin Baigent, Chair, Oxford University; Professor Alison D Murray, University of Aberdeen; Professor Gary Ford, Oxford University; Dr. Jonathan Emberson, Oxford University.

CONSORT 2010 checklist of information to include when reporting a randomised trial*

Section/Topic	Item No	Checklist item	Reported on page No
**Title and abstract**			
	1a	Identification as a randomised trial in the title	1
	1b	Structured summary of trial design, methods, results, and conclusions (for specific guidance see CONSORT for abstracts)	4
**Introduction**			
Background and objectives	2a	Scientific background and explanation of rationale	7
	2b	Specific objectives or hypotheses	7
**Methods**			
Trial design	3a	Description of trial design (such as parallel, factorial) including allocation ratio	Supplement p3
	3b	Important changes to methods after trial commencement (such as eligibility criteria), with reasons	N/a
Participants	4a	Eligibility criteria for participants	Supplement p2
	4b	Settings and locations where the data were collected	Supplement p3
Interventions	5	The interventions for each group with sufficient details to allow replication, including how and when they were actually administered	8

Outcomes	6a	Completely defined pre-specified primary and secondary outcome measures, including how and when they were assessed	8,9 and Suplement p3
	6b	Any changes to trial outcomes after the trial commenced, with reasons	N/a
Sample size	7a	How sample size was determined	Supplement p3
	7b	When applicable, explanation of any interim analyses and stopping guidelines	N/a
Randomisation:			
Sequence generation	8a	Method used to generate the random allocation sequence	8
	8b	Type of randomisation; details of any restriction (such as blocking and block size)	8
Allocation concealment mechanism	9	Mechanism used to implement the random allocation sequence (such as sequentially numbered containers), describing any steps taken to conceal the sequence until interventions were assigned	8
Implementation	10	Who generated the random allocation sequence, who enrolled participants, and who assigned participants to interventions	8
Blinding	11a	If done, who was blinded after assignment to interventions (for example, participants, care providers, those assessing outcomes) and how	8/9
	11b	If relevant, description of the similarity of interventions	N/a
Statistical methods	12a	Statistical methods used to compare groups for primary and secondary outcomes	10 and Supplement p5

	12b	Methods for additional analyses, such as subgroup analyses and adjusted analyses	Supplement p5
**Results**			
Participant flow (a diagram is strongly recommended)	13a	For each group, the numbers of participants who were randomly assigned, received intended treatment, and were analysed for the primary outcome	Supplement p6
	13b	For each group, losses and exclusions after randomisation, together with reasons	Supplement p6
Recruitment	14a	Dates defining the periods of recruitment and follow-up	Supplement p3
	14b	Why the trial ended or was stopped	Supplement p3
Baseline data	15	A table showing baseline demographic and clinical characteristics for each group	17 and supplement p9
Numbers analysed	16	For each group, number of participants (denominator) included in each analysis and whether the analysis was by original assigned groups	11 and Supplement p10-15
Outcomes and estimation	17a	For each primary and secondary outcome, results for each group, and the estimated effect size and its precision (such as 95% confidence interval)	11 and Supplement p10-15
	17b	For binary outcomes, presentation of both absolute and relative effect sizes is recommended	N/a

Ancillary analyses	18	Results of any other analyses performed, including subgroup analyses and adjusted analyses, distinguishing pre-specified from exploratory	11 and Supplement p10-15
Harms	19	All important harms or unintended effects in each group (for specific guidance see CONSORT for harms)	Supplement p3
**Discussion**			
Limitations	20	Trial limitations, addressing sources of potential bias, imprecision, and, if relevant, multiplicity of analyses	13
Generalisability	21	Generalisability (external validity, applicability) of the trial findings	12/13
Interpretation	22	Interpretation consistent with results, balancing benefits and harms, and considering other relevant evidence	12/13
**Other information**			
Registration	23	Registration number and name of trial registry	5
Protocol	24	Where the full trial protocol can be accessed, if available	8
Funding	25	Sources of funding and other support (such as supply of drugs), role of funders	14

*We strongly recommend reading this statement in conjunction with the CONSORT 2010 Explanation and Elaboration for important clarifications on all the items. If relevant, we also recommend reading CONSORT extensions for cluster randomised trials, non-inferiority and equivalence trials, non-pharmacological treatments, herbal interventions, and pragmatic trials. Additional extensions are forthcoming: for those and for up to date references relevant to this checklist, see www.consort-statement.org.
