# Supplementary material for: Effects of Cilostazol and Isosorbide Mononitrate on Cerebral Hemodynamics in the LACI-1 Randomized Controlled Trial
Source: Stroke. 2021 Dec 1;53(1):29–33. doi: 10.1161/STROKEAHA.121.034866 (PMC8700302; doi:10.1161/STROKEAHA.121.034866)
Supplement: Supplementary file 2 [file str-53-029-s002.pdf]

Change of Authorship Form

(Must be completed and signed by ALL authors)

Please check all that apply

_____New author(s) have been added (in addition to this form, all new authors must complete the copyright transfer agreement and conflict of interest disclosure.

_____Change in order of authorship.

✔ An author wishes to remove his/her name. An author's name may only be removed his/her own request and a letter signed by the author should accompany this form

Manuscript Number STROKE/2021/034866R1

Manuscript Title Effects of cilostazol and isosorbide mononitrate on cerebral haemodynamics in the LACI-1 randomised controlled trial

Former Authorship

Please list ALL AUTHORS in the same order as the original submission. For more than 12, use an extra sheet.

**Print Name**		**Print Name**	
Name (1)	Gordon W Blair	Name (7)	Iona Hamilton
Name (2)	Esther Janssen	Name (8)	Katie Flaherty
Name (3)	Michael S Stringer	Name (9)	Jason P Appleton
Name (4)	Michael J Thrippleton	Name (10)	Nikola Sprigg
Name (5)	Francesca Chappell	Name (11)	Fergus N Doubal
Name (6)	Yulu Shi	Name (12)	Philip M Bath

New Authorship

All authors must sign below agreeing to the changes in authorship. The authorship order must reflect the authorship order of the manuscript.

Name (1)	Gordon W Blair	Signature		Date	
Name (2)	Esther Janssen	Signature		Date	
Name (3)	Michael S Stringer	Signature		Date	
Name (4)	Michael J Thrippleton	Signature		Date	
Name (5)	Francesca Chappell	Signature		Date	
Name (6)	Yulu Shi	Signature		Date	
Name (7)	Iona Hamilton	Signature		Date	
Name (8)	Katie Flaherty	Signature		Date	
Name (9)	Jason P Appleton	Signature	[signature]	Date	11/08/2021
Name (10)	Fergus N Doubal	Signature		Date	
Name (11)	Philip M Bath	Signature		Date	
Name (12)	Joanna M Wardlaw	Signature		Date	

Please scan and email to stroke@strokeahajournal.org.
