# Supplementary material for: Effects of Cilostazol and Isosorbide Mononitrate on Cerebral Hemodynamics in the LACI-1 Randomized Controlled Trial
Source: Stroke. 2021 Dec 1;53(1):29–33. doi: 10.1161/STROKEAHA.121.034866 (PMC8700302; doi:10.1161/STROKEAHA.121.034866)
Supplement: Supplementary file 8 [file str-53-029-s008.pdf]

Change of Authorship Form

(Must be completed and signed by ALL authors)

Please check all that apply

_____ New author(s) have been added (in addition to this form, all new authors must complete the copyright transfer agreement and conflict of interest disclosure.

_____ Change in order of authorship.

✔ An author wishes to remove his/her name. An author's name may only be removed his/her own request and a letter signed by the author should accompany this form

Manuscript Number STROKE/2021/034866R1

Manuscript Title Effects of cilostazol and isosorbide mononitrate on cerebral haemodynamics in the LACI-1 randomised controlled trial

Former Authorship

Please list ALL AUTHORS in the same order as the original submission. For more than 12, use an extra sheet.

Print Name	Print Name
Name (1) Gordon W Blair	Name (7) Iona Hamilton
Name (2) Esther Janssen	Name (8) Katie Flaherty
Name (3) Michael S Stringer	Name (9) Jason P Appleton
Name (4) Michael J Thrippleton	Name (10) Nikola Sprigg
Name (5) Francesca Chappell	Name (11) Fergus N Doubal
Name (6) Yulu Shi	Name (12) Philip M Bath

New Authorship

All authors must sign below agreeing to the changes in authorship. The authorship order must reflect the authorship order of the manuscript.

Name	Signature	Date
Name (1) Gordon W Blair	Signature	Date
Name (2) Esther Janssen	Signature	Date
Name (3) Michael S Stringer	Signature	Date
Name (4) Michael J Thrippleton	Signature	Date
Name (5) Francesca Chappell	Signature	Date
Name (6) Yulu Shi	Signature	Date
Name (7) Iona Hamilton	Signature	Date
Name (8) Katie Flaherty	Signature K Flaherty	Date 8/8/21
Name (9) Jason P Appleton	Signature	Date
Name (10) Fergus N Doubal	Signature	Date
Name (11) Philip M Bath	Signature	Date
Name (12) Joanna M Wardlaw	Signature	Date

Please scan and email to stroke@strokeahajournal.org.
