# Supplementary material for: Effects of Cilostazol and Isosorbide Mononitrate on Cerebral Hemodynamics in the LACI-1 Randomized Controlled Trial
Source: Stroke. 2021 Dec 1;53(1):29–33. doi: 10.1161/STROKEAHA.121.034866 (PMC8700302; doi:10.1161/STROKEAHA.121.034866)
Supplement: Supplementary file 15 [file str-53-029-s015.pdf]

dallen@origineditorial.com

From: Bath Philip <Philip.Bath@nottingham.ac.uk>
Sent: Thursday, August 5, 2021 12:23 PM
To: WARDLAW Joanna
Cc: stroke@strokeahajournal.org
Subject: Re: STROKE/2021/034866R1 Decision Letter

Dear Joanna,

I have had a response - we should remove her from authorship. I write this has her academic line manager.

All the best,

philip

Philip M Bath FRCP DSc FMedSci
Stroke Association Professor of Stroke Medicine
Chief Investigator: PROTECT-CH, BEET-Winter, ENOS-2, SAVANNAH studies
Emeritus NIHR Senior Investigator
orcid.org/0000-0003-2734-5132

Visit https://www.protect-trial.net/

Stroke Trials Unit, Division of Clinical Neuroscience
University of Nottingham
Queen's Medical Centre
Nottingham NG7 2UH UK

Tel: +44 115 823 1768
Fax: +44 115 823 1767
Secretary: +44 115 823 1765
Web: www.nottingham.ac.uk/research/groups/stroke/

> On 4 Aug 2021, at 18:05, Philip Bath <mszpb@exmail.nottingham.ac.uk> wrote:
>
> Joanna,
>
> I do have a backdoor means of contacting Niki, just one I am trying not to use to protect her. Anyway, I have asked her.
>
> All the best,
>
> philip
>
>> On 3 Aug 2021, at 08:25, Philip Bath <mszpb@exmail.nottingham.ac.uk> wrote:
>>
>> Joanna,
>>
>> Assuming you want to use option 1 I can do/sign the 1c bit.

Philip

On 2 Aug 2021, at 21:28, stroke@strokeahajournal.org wrote:

August 2, 2021

Prof. Joanna M. Wardlaw
University of Edinburgh
Centre for Clinical Brain Sciences
Chancellor's Building
49 Little France Crescent
Edinburgh EH16 4SB
UNITED KINGDOM

MS ID#: STROKE/2021/034866R1
MS TITLE: Effects of cilostazol and isosorbide mononitrate on cerebral haemodynamics in the LACI-1 randomised controlled trial

Dear Prof. Wardlaw:

We are pleased to inform you that your manuscript has been accepted for publication in Stroke. We sincerely apologize for the long review period. The Editorial office staff has been behind on tasks.

We are sorry to learn Dr. Sprigg is unwell. There are two options forward with regard to authorship.
1) If Dr. Sprigg is to be kept as an author,
the corresponding author should submit:
a) Documentation of attempts to contact the contributing author and/or documentation that the author is unable to continue authorship for medical reasons.
b) A letter signed from the corresponding author that confirms that the contributing author fulfilled authorship criteria. The criteria are outlined in the Authorship Responsibility and Copyright Transfer Agreement form or
the Licensing Agreement form.
c) A letter signed by a senior member of the contributing author's institution that confirms that the work was carried out as described and confirms any conflicts of interest that the contributing author might have. That person would also sign the publication agreement and disclosure forms on behalf of Dr. Sprigg.

Option 2
2) If Dr. Sprigg is to be removed as an author, the corresponding author should supply these items:
a) All authors must confirm, in writing, the deletion. You may use the change of authorship form if convenient:
https://www.ahajournals.org/str/revised-accepted-manuscripts
b) A senior member of the contributing author's institution also must confirm, in writing, the deletion.

Please let us know if you have questions about these options.

Prior to sending the manuscript to the publisher, we ask that all material required for publication is submitted. Timely publication depends upon your compliance with these requirements. We cannot process your manuscript until all final documents have been received. Please review your submission for the following:

1. Funding Sources: All grant funding agency abbreviations should be completely spelled out, with the exception of the American NIH.

2. References:
a. Please update/complete references 4 and 17

b. Please review your reference list to ensure that all references are complete, up-to-date, and do not cite retracted or potentially problematic sources without proper context.

3. Please provide a clean, final Word file for the text.

4. Online Supplemental Data:
a. Please include the CONSORT checklist with the other supplemental material. We have started publishing checklist where available.

b. In the manuscript text, following the Acknowledgments, Sources of Funding, & Disclosures section, please include a list of the supplemental materials with a callout to any references that are in the Supplemental Material only, for example:

Supplemental Materials
Expanded Materials & Methods
Online Figures I-IV
Online Video I
Data Set

c. A single-spaced PDF of your supplemental data must be provided. The first page of this PDF should include the heading, "SUPPLEMENTAL MATERIAL." Please note that this single PDF should include all of the supplemental material related to your manuscript. Online supplemental data is NOT copy edited or typeset. It will be uploaded to the journal website as is.

5. Please note that the manuscript is over the word count limit. An excess word fee of $425 will apply in addition to the standard page charge.

The authors have selected the Open Access Agreement CC-BY publishing license for this article. This license must MEET THE NEEDS OF THE FUNDERS OF EVERY AUTHOR. Authors are responsible for paying the publication costs associated with the license selected. Information is available at https://www.ahajournals.org/open-access-information. If

you need to change the agreement selected, please contact the editorial office. If a change is made, all authors are required to complete the new forms before the manuscript can enter the production cycle. It is not acceptable to change agreements after the article is published.

You will be receiving an Open Access Publication Charge letter from the Journal's Publisher, Wolters Kluwer, when your manuscript is routed for copy editing. The letter will include instructions on how to submit any open access charges. The email will be from publicationservices@copyright.com with the subject line "Please Submit Your Open Access Article Publication Charge(s)." Please complete payment of the Open Access charges within 48 hours of receipt.

During the copy-editing phase, there may be some changes in phraseology, but there will be no alteration to scientific content. When you receive your galley proofs, please read, correct, and return them immediately so that publication will not be delayed.

Congratulations on this outstanding work and thank you for contributing to Stroke.

With kind regards,

Stephen Davis
Handling Editor

Marc Fisher, MD
Senior Guest Editor
Stroke

Submit your best science to #ISC22! Deadline Aug 24, 2021.

For Submission Info:
https://professional.heart.org/en/meetings/international-stroke-conference

This message and any attachment are intended solely for the addressee and may contain confidential information. If you have received this message in error, please contact the sender and delete the email and attachment.

Any views or opinions expressed by the author of this email do not necessarily reflect the views of the University of Nottingham. Email communications with the University of Nottingham may be monitored where permitted by law.
